# Cognitive Behavioral Therapy Management of a Patient with Atypical Anorexia Nervosa

**DOI:** 10.1155/2019/4736419

**Published:** 2019-10-10

**Authors:** Nisansala Liyanage, Chathurie Suraweera, Asiri Rodrigo

**Affiliations:** ^1^University Psychiatry Unit North Colombo Teaching Hospital, Ragama, Sri Lanka; ^2^Department of Psychological Medicine, University of Colombo, Sri Lanka; ^3^Department of Psychiatry, Faculty of Medicine, University of Kelaniya, Ragama, Sri Lanka

## Abstract

Eating disorders are becoming more common in nonwestern societies and some of these presentations are atypical variants such as atypical anorexia nervosa. There is very little data on how to treat these patients. This case study reports the treatment of a young adult female in Sri Lanka who presented with atypical anorexia nervosa and moderate depressive disorder. She was successfully treated with nine sessions of enhanced cognitive-behavioural therapy (CBT-E). According to our knowledge this is the first case report that describes the management of a patient with atypical anorexia nervosa using psychological therapy specifically adapted to nonwestern context.

## 1. Introduction

Prevalence of eating disorders, including anorexia nervosa (AN), in Asia is increasing [1]. Sri Lanka is no exception with clinicians encountering higher number of patients with eating disorders than before [2]. These patients are challenging to manage and suffer from significant physical and psychosocial impairment [3]. In spite of a clear need for treatment effective in managing eating disorders, only few options are available. The most effective treatment available is cognitive behavioural therapy (CBT) [4]. However, lack of evidence for efficacy of CBT for eating disorders in nonwestern settings, poor resource availability for such therapy and variant clinical presentations seen in Asian cultures which are different from Western presentations remain a challenge to using CBT in treating atypical variant of AN. Atypical AN is similar to classic AN in all other respects except for experiencing a fear of weight gain or becoming fat, and atypical form is rather common in Sri Lanka [2].

Fairburn et al. who proposed Enhanced CBT (CBT-E) questioned the view held by psychiatry taxonomies such as DSM and ICD, that different eating disorders are distinct conditions, each requiring their own form of treatment [5]. They argued that what is most striking about the eating disorders is not what distinguishes them but how much they have in common including the characteristic core psychopathology of eating disorders, the over-evaluation of the importance of food, shape and weight. In turn they proposed a transdiagnostic CBT that addresses the over-evaluation of shape and weight and preoccupation with thoughts about food and eating, and thereby can be used in all eating disorders [5]. While NFP-AN has preoccupation of food it does not have over-evaluation of shape. Therefore, effectiveness of CBT-E in NFP-AN is debatable.

## 2. Case Presentation

Miss J is a 24-year-old single student from Anuradhapura which is a rural area situated 250 km away from the capital of Sri Lanka. She presented with a history of reduced oral intake for 3 years with malaise and fatigability. Her reduced food intake started while she was studying for the General Certificate of Education Advanced Level examination which is one of the most competitive examinations in Sri Lanka. Initially she thought that reduced food intake would help her to study well by reducing drowsiness in the night and increasing attention span in the day time. Miss J developed abdominal discomfort including fullness of stomach and belching after meals following reduced food intake for few months. Further, she lost 18 kg (36% of total body weight) over a period of three years since reduction of food intake. In the last 3 months, Miss J completely refused solid meals and consumed liquid meals only as she could not tolerate the appearance and smell of solid foods. She was preoccupied about food which led to negative emotions and distracted her from studies. Miss J developed depressive symptoms 1 year after she restricted food intake in the context of her inability to enter University. Miss J started to question her intellectual ability and had poor self-evaluation. She developed amenorrhea 2 years after her initial symptoms. As Miss J was concerned about her excessive weight loss and being very thin, she attempted to make changes in her dietary habits. However, anxiety and abdominal symptoms prevented her from eating when she tried doing so. There was no history of binge-eating, self-induced vomiting, abuse of slimming medications, excessive exercise, diarrhoea, constipation or vomiting. She had no significant developmental history or features suggestive autism such as restrictive repetitive interests, difficulties in social relationships, social conversation or interpretation of others emotions.

After excluding possible organic causes for her presentation, Miss J was referred to the psychiatric unit for further evaluation where she was diagnosed to have avoidant/restrictive food intake disorder or Atypical AN-non fat-phobic type with co morbid moderate depressive episode. On admission Miss J was started on mirtazapine 7.5 mg nocte. Following week enhanced CBT was commenced. Inputs from dietician and gastroenterologist were sought.

## 3. Treatment

The initial CBT sessions were conducted while she was an inpatient due to the logistical difficulties of travelling more than 250 km for treatment. Therapy began with psychoeducation about eating disorders and CBT-E and its efficacy in the treatment of these disorders, after which Miss J expressed her willingness to engage in the therapy. This was further strengthened when the CBT conceptualization of her presentation was shared with Miss J. She believed that it is relevant to her and “makes sense”. Potential problems with attending for psychotherapy such as having to travel from Anuradhapura for sessions were discussed and resolved. At the outset of treatment, severity of Miss J's eating disorder and depressive disorder and level of functioning were assessed using validated versions of Eating Attitude Questionnaire (EAT-26), Beck Depression Inventory (BDI II) and he Global Assessment of Functioning (GAF). Higher scores of EAT-26 and BDI II indicate severe forms of eating disorder and depression respectively and higher GAF score point to better functioning.

The next step was strengthening her resolve to change the dietary habits. Negative effects of food restriction and being underweight were discussed. Miss J was then advised to make a list of reasons to eat more and gain weight which was used during therapy to maintain the commitment. Miss J was initially reluctant to change her eating behaviors citing abdominal discomfort when she ate more. However, when the discrepancy between her current food restricting behaviour and its deleterious effects on achieving her future academic goals were pointed out, Miss J expressed her wish to change. Principles of motivational interviewing were adopted to engage her further. Once her commitment to therapy and change of dietary habits were established, CBT model was further explained to her. The interplay of environment, thoughts, emotions, behaviours, and physiology was discussed taking examples from her presentation and how this maintained the restrictive dietary habits was explained.

**Figure 1:**
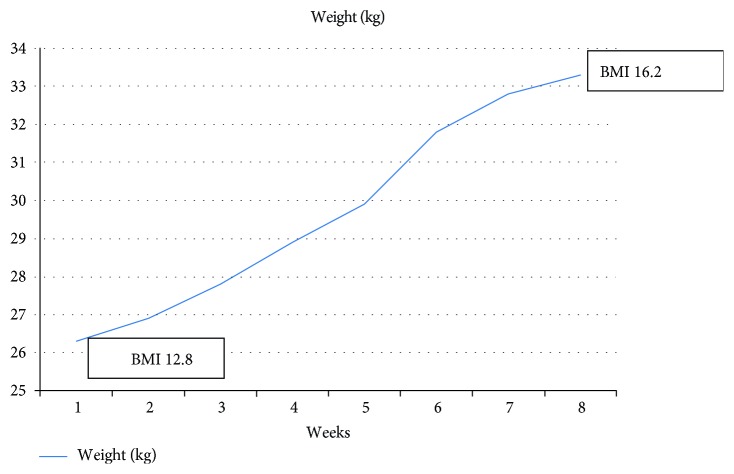
Progress of weight over the course of therapy.

CBT-E requires weekly in-session weighing. The rationale for this was explained to Miss J. Her weight and BMI were plotted weekly on a graph (Figure 1) and because weight naturally fluctuates from week to week, the graph was reviewed in detail with Miss J every 2 weeks with an emphasis on the overall trend in change of weight.

Initial goal of CBT for Miss J included establishing a normalised pattern of eating. This pattern was collaboratively agreed as eating three main meals and three small meals per day, spaced no longer than 4 hours apart. Initially the goal was simply for her to eat in a patterned way at specified times without focusing on what she ate. When a more normalised pattern of eating became established the focus was shifted to the content of her meals. Relaxation techniques were used to address anxiety and sensations of fullness associated with hunger. Distraction methods used to avoid tendency to have a snack. She was exposed to smell of food at the beginning, as the first step of graduated exposure. She was advised to adhere to the pattern under all circumstances without eating outside the agreed schedule. Although it was difficult for her at the beginning, after a few weeks Miss J was able to adopt an automatic, normalised pattern of eating. A list of avoided foods was generated, and these foods were then placed into three groups. Group 1 foods were those least feared, and Group 3 foods were those most feared. Using graduated exposure, these foods were introduced into her diet in small portions, beginning with Group 1 and progressing to Group 3. It was demonstrated to her that she could have heavy food without getting abdominal discomfort and anxiety.

Miss J was asked to keep a real-time daily food record including time, portion size and content of meals as well as emotions, thoughts, behaviours, and significant events associated with meals on each day. She was asked to focus on physiological sensations and whether she thought what she ate was excessive. The importance of completing the record real time and rationale for keeping the records was discussed with her focusing on her ability to have control over things, which was one of her personal goals. Miss J kept records reliably, which was appreciated and reinforced at each session.

In addition to establishing a normal eating pattern, we helped Miss J to engage in enjoyable and meaningful activities she was not able to do over the last 3 years. She generated a list of enjoyable and meaningful activities in which she engaged in the past as well as new activities that she would like to try. Each week, she engaged in at least one to two new activities. Because Miss J wanted to complete her studies and find meaningful employment she was encouraged to work towards those goals. She recorded these activities on monitoring forms, and they were discussed in sessions each week. By the end of treatment, Miss J was able to study and she applied for suitable jobs.

Finally, near the end of treatment, her perfectionism was addressed. Origins of her perfectionism were explored and it was explained to Miss J that this was a maintaining factor of her eating disorder. As an example of her perfectionistic thinking, she believed that she was a “failure” if she was not able to study for 10 hours a day. Miss J was able to challenge and change such irrational thoughts or beliefs through cognitive restructuring. She was assisted to generate a list of her positive self-qualities to redirect her from overvaluing eating patterns and achievement and learn to value other important aspects herself. She was aided in restructuring her life goals to be more rational, as some of the goals she had set for herself were unrealistic. For example, she believed the only way to achieve success in higher education was through entrance to a state University after being successful at the Advanced Level examination and the only way to be successful in life was by working in a government institution. These goals were restructured as getting higher education from any recognised University and working in a meaningful job whether in private or public sector.

At the end of treatment, Miss J was educated about how to maintain the changes she had made and prevent relapse. Future situations which may trigger eating disorder symptoms and perfectionism were identified such as final exam or starting a new job. She was empowered by the knowledge that she had learnt the tools she needed to maintain the changes she had made.

First 5 psychotherapy sessions were conducted weekly while she was an inpatient. She was discharged after 5 weeks hospital stay when her feeding habits were normalised. Next four sessions were conducted fortnightly as an outpatient. EAT-26, BDI II and GAF score improved markedly from pre-treatment scores of 19, 16, and 40 to post treatment scores of 12, 11, and 70 respectively indicating significant improvement of symptoms of the eating disorder and depressive disorder as well as functionality with treatment. She reported modest improvement of her abdominal symptoms and gained 7 kg over 2 months.

## 4. Discussion

This case report suggests enhanced cognitive behavioural therapy is effective for atypical presentations of eating disorders in a nonwestern setting. Miss J presents with restrictive eating, the hallmark diagnostic feature of anorexia nervosa. However, in her case restrictive eating is not driven by fear of weight gain which is known as nonfat phobic anorexia nervosa. Individuals with NFP-AN typically report an awareness of their low weight status and a desire to gain weight and often report other reasons such as gastrointestinal difficulties for their food restriction as in Miss J [6]. This is similar to DSM V diagnosis of avoidant/restrictive food intake disorder (ARFID) in which the restrictive food intake is driven by sensitivity to the sensory characteristics of food, fear of aversive consequences of eating, and/or lack of interest in eating or food [7]. However, some argue that ARFID is different from NFPAN in that individuals with implicit associations of NFP-AN were inconsistent with their explicit endorsements, possibly reflecting a minimizing response style on explicit measures in contrast to implicit associations being consistent with explicit endorsement in ARFID [8]. Enhanced CBT (CBT-E) developed by Fairburn et al. was used in her treatment [9, 10]. While CBT-E is the most widely used psychological therapy for eating disorders including anorexia nervosa [4], to the best of our knowledge this is the first report of using CBT-E for NFP-AN or ARFID. It is noteworthy that this treatment was completed in 9 sessions and conducted in a busy clinical setting with low human resources. This suggests CBT-E is truly a transdiagnostic and transcultural therapy.

One of the main reason for CBT-E to be successful in Miss J is the ability to tailor it specifically to the patient's presentation. Fairburn recommend adjusting CBT-E according to the patient by using additional CBT techniques to suit individual patient's need [9]. Broad form of the treatment (CBT-Eb) is used in her treatment to addresses external obstacles to change such as perfectionism and low self-esteem, in addition to the core eating disorder psychopathology. This treatment successfully addressed anxiety, somatic symptoms associated with meals, perfectionism, need for control projected to food restriction, lack of meaningful activities and poor perceived self worth which acted as maintaining factors of the eating disorder.

CBT-E typically involves 20 sessions for people who are not significantly underweight and up to 40 sessions for those who are underweight [9]. Out of 20 sessions first 8 sessions are dedicated for engagement, psychoeducation, introduction to a meal plan followed by 10 sessions involving one or more of CBT modules that address body image, dietary restraint and influences of mood and events over eating. In last few sessions patients consolidate treatment gains and plan for the future. Eating disorder of Miss J, a significantly underweight patient, was successfully treated with far less number of sessions than 40. This could be explained by her willingness to readily engage in therapy, not having to address body distortions, probable greater role of medication in the treatment and facilitation of psychological treatment during her inpatient stay. Miss J reported that her treating doctors and nursing staff in the ward reiterated the psychological principles she learned in the therapy during their daily rounds and 1 : 1 sessions although they did not introduce new principles. The hospital staff was instrumental in establishing the meal plan. The role played by hospital staff may have undoubtedly lead to the expeditious recovery. Lack of insight and low motivation to change has been well documented as treatment barriers in eating disorders [11]. This is reflected in CBT-E by dedicating many sessions for engagement [9]. The fact that Miss J was insightful and motivated to change may have contributed to the rapid improvement of symptoms.

Most important cultural changes to CBT-E in this patient were adopting more directive or instructional style and putting more emphasis on somatic symptoms, and the former may have made the therapy shorter.

Though this case study provides support for the utilization of CBT-E for nonwestern patients with eating disorders even when they present with atypical variant of eating disorder, the result cannot be generalised. Further research studies, specially randomized controlled trials with adequate number of participants, are needed to establish the efficacy of CBT-E for these patients.
